# Monitoring Circulating Myeloid Cells in Peritonitis with an In Vivo Imaging Flow Cytometer

**DOI:** 10.3390/biom14080886

**Published:** 2024-07-23

**Authors:** Sunitha Pulikkot, Souvik Paul, Alexxus Hall, Brianna Gardner, Wei Liu, Liang Hu, Anthony T. Vella, Yunfeng Chen, Zhichao Fan

**Affiliations:** 1 Department of Immunology, School of Medicine, UConn Health, 263 Farmington Ave., Farmington, CT 06030, USA; 2 Department of Biochemistry and Molecular Biology, University of Texas Medical Branch, 301 University Blvd, Galveston, TX 77555, USA; 3 Department of Pathology, University of Texas Medical Branch, 301 University Blvd, Galveston, TX 77555, USA; 4 Academy of Integrative Medicine, Shanghai University of Traditional Chinese Medicine, 1200 Cai Lun Road, Shanghai 201203, China

**Keywords:** in vivo flow cytometry, neutrophils, peritonitis

## Abstract

Peritonitis is a common and life-threatening inflammatory disease. Myeloid cells are elevated in the peripheral blood and contribute to peritonitis, but their circulating dynamics are not clear. In vivo flow cytometry (IVFC) is a noninvasive technique for monitoring the dynamics of circulating cells in live animals. It has been extensively used to detect circulating tumor cells, but rarely for monitoring immune cells. Here, we describe a method adapting an intravital microscope for IVFC so that we can monitor LysM-EGFP-labeled circulating myeloid cells in a tumor necrosis factor (TNF) α-induced peritonitis mouse model. Using this IVFC method, we quantified the blood flow velocity and cell concentration in circulation. We observed a significant increase in LysM-EGFP+ cells in circulation after TNFα intraperitoneal (i.p.) injection, which reached a plateau in ~20 min. Conventional cytometry analysis showed that most LysM-EGFP+ cells were neutrophils. Increasing blood neutrophils were accompanied by neutrophil recruitment to the peritoneal cavity and neutrophil emigration from the bone marrow. We then monitored neutrophil CD64 expression in vivo and found a significant increase in TNFα-induced peritonitis. We also found that CD18 blockade doubled the circulating neutrophil number in TNFα-induced peritonitis, suggesting that CD18 is critical for neutrophil recruitment in peritonitis. Overall, we demonstrate that IVFC techniques are useful for studying the circulating dynamics of immune cells during inflammatory diseases.

## 1. Introduction

Peritonitis is the inflammation of the peritoneum, the membrane that lines the inner abdominal wall and encloses organs within the abdomen. Peritonitis is life-threatening and may lead to multiple organ failure and sepsis [1,2,3,4], and has significant morbidity and mortality rates that are reported to be between 13 and 43% [5]. It is associated with the host’s systemic inflammatory response in response to multiple clinical conditions such as trauma, surgery [6,7], liver cirrhosis [8], peritoneal dialysis catheters [9], or disruption to the gastrointestinal tract [6,10]. Along with early diagnosis and prevention, understanding different variables and their influence on peritonitis is required [1,5]. Neutrophils are the most abundant leukocytes in human blood and are critical for innate immunity and inflammation [11,12]. Neutrophil infiltration is a hallmark of peritonitis, and might be induced by the i.p. injection of CXCL1 [11,13,14] or TNF α [15,16,17]. Neutrophils are also elevated in peripheral blood and contribute to peritonitis [18,19,20], so understanding how neutrophil dynamics in the blood change over time may improve our understanding of how they impact peritonitis. Conventional methods to count neutrophils are limited by single-time-point sampling. In vivo flow cytometry (IVFC) enables us to monitor the circulating cells in the bloodstream in a real-time scenario without invasive methods or sacrificing the animal [21,22,23,24,25,26,27,28]. Most IVFC studies monitored circulating tumor cells (CTCs) to study cancer metastasis. A few IVFC studies focus on circulating immune cells, including regulatory T cells in the allograft response [27], the interaction of circulating dendric cells with CTCs [29], CX3CR1-EGFP-labeled monocytes, and LysM-GFP-labeled neutrophils [30]. However, these studies require custom-built instruments, limiting their use in most biomedical research laboratories.

Since neutrophil dynamics might provide essential information for disease progression and IVFC can monitor neutrophil dynamics non-invasively, here, we describe a method that adapts an intravital microscope to an IVFC and uses it to monitor LysM-EGFP-labeled circulating myeloid cells (mainly neutrophils) in a TNFα-induced peritonitis mouse model [20]. Compared with existing methods, our method is based on scanning microscopes, such as confocal or multi-photon microscopes, which are available in most core facilities of biomedical research institutions.

## 2. Materials and Methods

### 2.1. Reagents

Phycoerythrin (PE)-conjugated rat anti-mouse Ly6G mAb, PE-conjugated rat anti-mouse CD64 mAb, Brilliant Violet 650 (BV650)-conjugated rat anti-mouse Ly6G mAb, and recombinant TNF-α were purchased from BioLegend (San Diego, CA, USA). Phosphate-buffered saline (PBS) was purchased from Gibco (Grand Island, NY, USA). Paraformaldehyde (PFA) was purchased from Sigma Aldrich (St. Louis, MO, USA). Rhodamine B-labeled dextran (70,000 MW) was purchased from Invitrogen (Waltham, MA, USA).

### 2.2. Mice

LysM-EGFP or Lyz2-EGFP mice [31] were originally obtained from Albert Einstein College of Medicine, and the C57BL/6 background LysM-EGFP mice were obtained from Dr. Klaus Ley from the La Jolla Institute for Immunology (currently at Augusta University) through material transfer agreements. Mice were fed a standard rodent chow diet and were housed in microisolator cages in a pathogen-free facility in the Center for Comparative Medicine at UConn Health. All experiments followed the UConn Health Institutional Animal Care and Use Committee (IACUC) guidelines, and approval for the use of rodents was obtained from the UConn Health IACUC according to criteria outlined in the Guide for the Care and Use of Laboratory Animals from the National Institutes of Health. Both male and female mice aged from 12 to 16 weeks and weighing above 20 g were used in this study. All experimental protocols were approved by the UConn Health Animal Care and Use Committee.

### 2.3. IVFC

**Figure 1:**
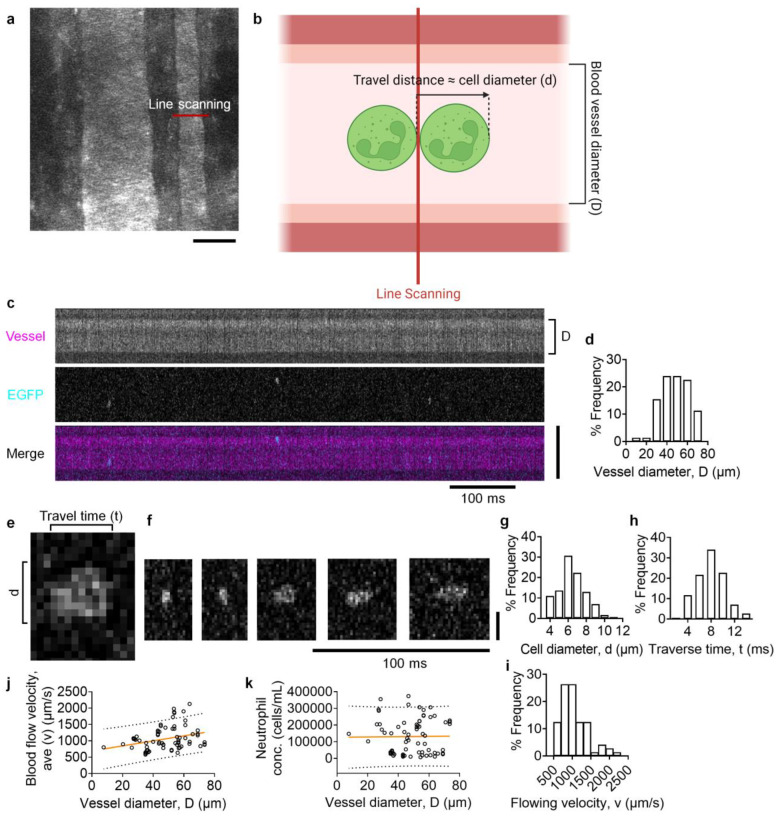
Monitoring of LysM-EGFP+ leukocytes by multi-photon imaging IVFC. (**a**) A representative multi-photon image of blood vessels labeled with Rhodamine B-labeled dextran in a mouse ear. A red line shows a representative line scanning position. The scale bar is 50 μm. (**b**) Schematics showing the transport of leukocytes through the line scanning. The cell traversing distance is similar to the cell diameter (d). (**c**) Representative line scanning images showing the diameter (D) of blood vessels labeled with Rhodamine B-labeled dextran (upper panel, magenta in the lower panel) and LysM-EGFP+ leukocytes (middle panel, cyan in the low panel) traversing the line scanning. The scale bar is 50 μm. The time bar is 100 ms. (**d**) A histogram depicting the frequency distribution of vessel diameters. n = 70 individual records of 7 mice. (**e**) A representative line scanning image showing the LysM-EGFP+ cell diameter (d) and traverse time (t) of cells traversing the line scan. (**f**) Five representative line scanning images showing LysM-EGFP+ leukocytes with different traverse times (t) transversing the line scanning. Fast to slow cells are shown from left to right. The scale bar is 10 μm, and the time bar is 100 ms. (**g**–**i**) Histograms showing the frequency distribution of the cell diameter (d, (**g**)), traverse time (t, (**h**)), and moving velocity (v, (**i**)) of LysM-EGFP+ leukocytes. n = 300 cells from 60 records of 6 mice. (**j**,**k**) The blood flow velocity (**j**) or neutrophil concentration (**k**) as a function of vessel diameter. The linear correlation (orange) and 90% prediction bands (dotted) were shown. n = 70 vessels from 7 mice.

LysM-EGFP mice were anesthetized with an intraperitoneal (i.p.) injection of ketamine hydrochloride (125 mg/kg) and xylazine (12.5 mg/kg) and were retro-orbitally injected with 20 μL 10 mg/mL Rhodamine B-labeled Dextran (70,000 MW) to outline the microvasculature. In the experiments measuring neutrophil CD64 expression in vivo, instead of Rhodamine B-labeled Dextran, 100 μL of 50 μg/mL PE-conjugated rat anti-mouse CD64 mAb was retro-orbitally injected to label CD64 on circulating cells and outline the microvasculature. In the CD18 blockade experiment, 100 μg CD18 blockade mAb or isotype control was retro-orbitally injected together with Rhodamine B-labeled Dextran. Then, mice were fixed on the stage of a Bruker’s upright multi-photon microscope (#4269) equipped with a Mai Tai High-Performance Ti:sapphire femtosecond pulsed laser (tuning range 690–1020 nm, set to 780 nm excitation in this assay), and mouse ear microvasculature was imaged (Figure 1a) using a 20× NA 0.95 water immersion objective. Line scanning was performed on vessels (artery or vein) with a diameter of 20–45 μm at ~650–850 Hz to enable IVFC measurement recording for all traversing LysM-EGFP+ leukocytes. We recorded 120 s for each time point. Thirty min after our first recording, mice were i.p. injected with TNFα (0.5 µg in 200 µL PBS) or vehicle control (PBS). IVFC measurement was performed 10, 20, 30, 60, 90, 120, 150, and 180 min after the injection. Line scanning images were analyzed by FIJI-ImageJ v2.0 to obtain cell diameter (d), traversing time (t), and blood vessel diameter (D). The number of cells (n) in each record was also counted.

### 2.4. Automated Data Analysis

Automated data analysis was achieved using Python (Python Software Foundation, Wilmington, DE, USA), with the code available in GitHub git@github.com:sodgp/cell_image_detection.git. The code processes the images to identify cells and extract relevant information such as cell dimensions and intensity values. Three functions are defined. The first two functions are used to calculate the diameter of the blood vessel and parse an XML file to extract the scan rate, respectively, and then the third function detects the cells in the image and retrieves their properties, such as dimensions and intensity values.

In the third function, image preprocessing was first carried out to extract the grayscale information from the RGBA color-formatted images, which then underwent segmentation processing. Thresholding was performed with the first image loaded into the program, rendering a threshold value for separating cells (brighter) from all non-cell particles in the background (darker). This threshold could be applied to all following images loaded into the program, which would be automatically adjusted based on each image’s brightness. Finally, a label map was applied to the binary image to mark each cell, with the assigned number and the cell’s measured length along the flow direction annotated next to the cell’s image.

### 2.5. Flow Cytometry

LysM-EGFP mice were i.p. injected with TNFα (0.5 µg) or vehicle control (PBS). One hour after the TNFα injection, mice were anesthetized with an i.p. injection of ketamine hydrochloride (125 mg/kg) and xylazine (12.5 mg/kg). Blood was collected using retro-orbital bleeding. Then, mice were euthanized using cervical dislocation. Bone marrow cells from one tibia and one femur were harvested and resuspended in 50 μL PBS. Then, 5 mL PBS was injected i.p., and peritoneal lavage was collected and resuspended in 50 μL PBS. Next, 50 μL blood, all bone marrow cells, and all peritoneal lavage cells were incubated with Ly6G-PE antibody (2 μg/mL) for 30 min at 4 °C, and then fixed with 1% PFA for 10 min. Red blood cells were lysed with deionized water for 30 s (stopped by adding 10× PBS). Leukocyte fluorescence was assessed with an LSRII (BD Biosciences, San Jose, CA, USA) and analyzed with FlowJo software (version 10.6).

To assess CD64 expression, 50 μL blood was collected through retro-orbital bleeding before and after TNFα (0.5 µg) or vehicle control (PBS) i.p. injection on C57BL/6 mice. Blood samples were incubated with 2 μg/mL PE-conjugated rat anti-mouse CD64 mAb and 2 μg/mL BV650-conjugated rat anti-mouse Ly6G mAb for 30 min at 4 °C, and then fixed with 1% PFA for 10 min. Red blood cells were lysed with deionized water for 30 s (stopped by adding 10× PBS). Leukocyte fluorescence was assessed with an LSRII (BD Biosciences, San Jose, CA, USA) and analyzed with FlowJo software (version 10.6).

### 2.6. Statistics

Statistical analysis was performed using PRISM software (version 8.30, GraphPad Software). Data analysis was performed using the unpaired two-sided *t*-test or two-way ANOVA test with Tukey’s multiple comparison correction, which is indicated in the Figure Legends, assuming a normal distribution of data. *p* values less than 0.05 were considered significant.

## 3. Results

### 3.1. Quantifying Neutrophil Blood Concentration Using Multi-Photon IVFC

IVFC is a powerful tool for quantifying the circulating cells in the bloodstream and is very useful in studying blood cell kinetics. Conventional fluorescence IVFC setups use a cylindrical lens to make a laser slit across the vessel and detect the epifluorescence signaling from the traversing cells using a photomultiplier tube (PMT) detector [21]. Unlike the conventional IVFC, we adapted a multi-photon intravital microscope to an IVFC by setting up ultrafast line scanning across the vessel. Similar methods have been used to detect CTCs and monocytes [27,31]. We extended the method to quantify the blood flow velocity and cell concentration in circulation (Figure 1). Specifically, Rhodamine B-labeled dextran (70,000 MW, Invitrogen) was retro-orbitally intravenously (i.v.) injected into LysM-EGFP mice to outline the microvasculature. Mouse-ear microvasculature was imaged (Figure 1a) using a Bruker’s upright multi-photon microscope (#4269) equipped with a Mai Tai High-Performance Ti:sapphire femtosecond pulsed laser (tuning range 690– 1020 nm, set to 780 nm excitation in this assay) and a 20× NA 0.95 water immersion objective. Both the artery and vein are visible in the image. Line scanning was conducted on vessels (artery or vein) with a diameter ranging from 20 to 75 μm at ~650–850 Hz (lines per second, depending on vessel diameter) to record all traversed LysM-EGFP+ leukocytes. When cells traverse the line scan, the EGFP signal is recorded, as illustrated in the schematics (Figure 1b). A typical line-scanning image is shown in Figure 1c. In this ~740 ms record, 3 LysM-EGFP leukocytes traversed the line scanning. From this kind of line-scanning data, we can easily obtain vessel diameters (D, Figure 1b–d) based on the Rhodamine B channel (the top panel of Figure 1c) and the cell diameters (d, Figure 1b,c,e–g) based on the EGFP channel (the middle panel of Figure 1c,e,f). In the line scanning image, the shape of the cells represents their traverse time (t) through the line scanning (Figure 1c,e,f). Our records showed that the traverse time is ~7.98 ± 2.44 ms (Figure 1h). Flowing leukocytes are mostly round [32,33]. Thus, when traversing through the line scanning, the traversing distance of a leukocyte is approximately equal to the cell diameter (d, Figure 1b). Our records show that LysM-EGFP+ leukocytes are ~6.6 ± 1.4 μm (Figure 1g), which is less than the previously reported neutrophil diameter (6.4–13.7 μm from different reports) [34,35]. This might be due to a higher autofluorescence background in mouse ears. However, this will not affect our calculation of moving velocity (v) because the measurements of time (t) and distance (d) are similarly influenced by this autofluorescence background. From above, we can estimate the moving velocity (v) of leukocytes using the equation v = d/t, which is ~1063 ± 357 μm/s (Figure 1i). By averaging v in one time point record, we can estimate the average blood flow velocity, ave (v). As said above, we can obtain the vessel diameter (D) from the Rhodamine B channel (Figure 1b–d). We also found that the blood flow velocity (v) is linearly correlated with the vessel diameter (D, Figure 1j). An F test showed that the slope is significantly non-zero (F = 6.792, *p* = 0.0112). A Pearson correlation coefficient assay showed that they were positively correlated (r = 0.2974, *p* = 0.0112).

In the total scanning time (T), which is 120 s in our record of each time point, we can count the LysM-EGFP+ leukocyte number (n). Thus, we can estimate the cell blood concentration using the two equations below:

Blood volume (V) = π (D/2)^2^ × ave (v) × T (1)

The concentration of LysM-EGFP + leukocytes = n/V(2)

**Figure 2:**
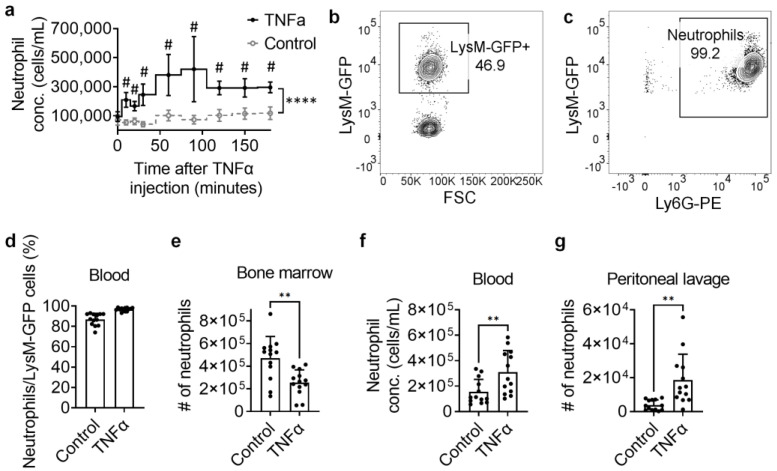
Neutrophil dynamics in TNFα-induced peritonitis. (**a**) Dynamic circulating concentrations of LysM-EGFP+ leukocytes (mostly neutrophils) within 3 h after TNFα or vehicle *i.p.* injection monitored by IVFC. IVFC measurements were performed at minutes 0, 10, 20, 30, 60, 90, 120, 150, and 180. Mean ± SEM. n = 6 mice. **** *p* < 0.0001 by the two-way ANOVA test. ^#^ *p* < 0.05 by multiple unpaired *t*-tests. (**b**,**c**) A representative gating of conventional flow cytometry measurements showing the percentage of LysM-GFP+ cells in blood leukocytes (**b**) and Ly6G+ neutrophils in LysM-GFP+ cells (**c**) from a mouse injected with TNFα for 1 h. (**d**) Conventional flow cytometry measurement showing percentages of Ly6G+ neutrophils in blood LysM-EGFP+ leukocytes at 1 h after TNFα or vehicle *i.p.* injection. Mean ± SD. n = 13 mice from 5 individual experiments. (**e**–**g**) Conventional flow cytometry measurement showing Ly6G+ neutrophil number in bone marrow (**e**), concentration in blood (**f**), and number in peritoneal lavage (**g**) from mice 1 h after TNFα or vehicle *i.p.* injection. Mean ± SD. n = 13 mice from 5 individual experiments. ** *p* < 0.01 by unpaired *t*-tests.

Using this estimation, we calculated that the concentration of LysM-EGFP+ leukocytes in unchallenged mice is ~13.1 ± 10.4 × 10^4^ cells/mL (Figure 1k and Figure 2a). This concentration is lower than the mouse neutrophil blood concentration in previous reports using Coulter counters [36,37], which might be due to the higher absorption of signal fluorescence and a higher autofluorescence background to reduce detection sensitivity. We noted that the neutrophil concentration is not correlated with the diameter of the blood vessels (Figure 1k). The F test showed that the slope is not significantly non-zero (F < 0.01, *p* = 0.923). The Pearson correlation coefficient assay showed that they were not correlated (r = 0.0117, *p* = 0.923). This might be because all the vessels we chose were less than 80 μm in diameter, which is within the penetration depth and the depth of field of multi-photon microscopy. Using conventional flow cytometry, we have confirmed that most (>90%) of the LysM-EGFP+ leukocytes are Ly6G+ neutrophils (Figure 2b).

### 3.2. Neutrophil Dynamics in TNFα-Induced Peritonitis by IVFC

Upon peritoneal inflammatory response induced by *i.p.* injection of TNFα, real-time dynamic changes in neutrophil concentration were monitored by IVFC and compared with controls injected with vehicle (Figure 2a). Neutrophil concentration was similar at the beginning of the assay in both control and TNFα-injected mice. Control mice showed a constant trend in the concentration within 3 h, whereas TNFα-injected mice showed a continuous increase in neutrophil concentration in the first 60 min after TNFα injection. The neutrophil concentration was significantly increased by around twice in the peripheral blood of TNFα-injected mice compared to controls (Figure 2a). It reached a plateau of ~4 × 10^5^/mL in TNFα-injected mice at minute 60 (Figure 2a). Compared with that before TNFα injection, neutrophil concentration significantly increased at minutes 30, 60, 90, 120, 150, and 180 (*p* = 0.049, 0.031, 0.028, 0.005, 0.012, and 0.003, respectively, unpaired *t*-tests) after TNFα injection. To confirm our IVFC data, we performed conventional flow cytometry to assess the Ly6G+ neutrophil number changes in bone marrow (BM), peripheral blood, and peritoneal lavage 1 h after TNFα injection (Figure 2c–e). Similar to our IVFC results, we saw that the neutrophil concentration increased by around twice in TNFα-injected mice compared to controls (Figure 2d). The concentration we observed using conventional flow cytometry (3.1 ± 1.7 × 10^5^ cells/mL) was ~20% less than when using IVFC (3.8 ± 2.8 × 10^5^ cells/mL). This might be due to the sample loss during preparation, especially during centrifugation and red blood cell lysis, and the dead volume in the flow cytometer. Thus, IVFC might be more accurate than conventional flow cytometry in counting exact blood cell numbers. However, we also noted that IVFC showed a lower neutrophil count compared to some hematology assays with lower dead volume and without red blood cell lysis, such as Coulter counters [36,37]. Consistent with the blood neutrophil count data, BM neutrophils were significantly decreased in TNFα-injected mice compared to controls (Figure 2c), suggesting that the increased blood neutrophils came from BM. As expected, neutrophils in peritoneal lavage were significantly increased in TNFα-injected mice compared to controls (Figure 2e).

### 3.3. In Vivo Measuring of CD64 Increase on Neutrophils in TNFα-Induced Peritonitis

**Figure 3:**
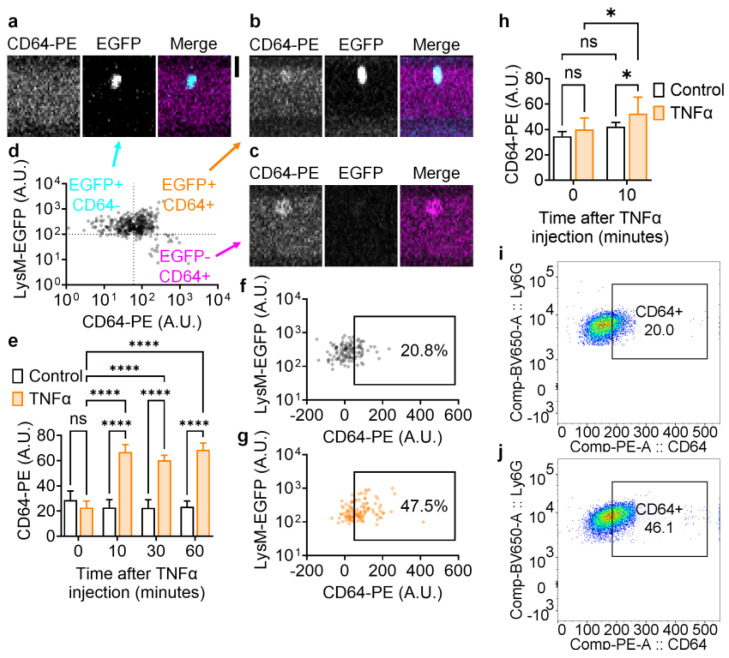
Neutrophil CD64 expression in TNFα-induced peritonitis. (**a**–**c**) Representative line scanning images showing GFP+CD64− (**a**), GFP+CD64+ (**b**), and GFP−CD64+ (**c**) leukocytes in circulation. (**d**) Dot plot of two-color IVFC recorded cells. N = 507 cells from 7 mice. (**e**) CD64-PE mean fluorescent intensity of circulating LysM-EGFP+ leukocytes (mostly neutrophils) in mice with TNFα or vehicle i.p. injection monitored by IVFC. Mean ± SEM. n = 150 cells from 5 mice. **** *p* < 0.0001 according to the two-way ANOVA followed by Tukey’s multiple comparison test. (**f**,**g**) Dot plots showing the percentage of CD64-PE+ cells in LysM-EGFP+ leukocytes before (**f**) and 10 min after (**g**) TNFα injection. n = 120 cells from 4 mice. (**h**) CD64-PE mean fluorescent intensity of blood neutrophils (Ly6G+) in mice with TNFα or vehicle i.p. injection assessed by conventional flow cytometry. The background mean fluorescence intensity of the PE channel was evaluated by Ly6G-BV650 mAb single-stained beads (~127) and substrated from the neutrophil PE mean fluorescent intensity. Mean ± SD. n = 6 mice. * *p* < 0.05 according the two-way ANOVA followed by Fisher’s LSD multiple comparisons test. (**i**,**j**) Representative dot plots showing the percentage of CD64-PE+ cells in neutrophils (Ly6G+) before (**i**) and 10 min after (**j**) TNFα injection. The gating boundary of the PE channel is based on Ly6G-BV650 mAb single-stained beads.

CD64, or Fc γ Receptor I, is a neutrophil activation marker, and its upregulation has been linked with peritonitis and sepsis [38,39]. We monitored CD64 expression in vivo by injecting PE-conjugated CD64 antibodies into LysM-EGFP mice (Figure 3). We observed CD64− and CD64+ neutrophils (CD64−EGFP+, Figure 3a, CD64+EGFP+, Figure 3b) as well as CD64+ non-neutrophil leukocytes (CD64+EGFP−, Figure 3c) in the circulation, which can be seen in the dot plot (Figure 3d). After TNFα injection, the neutrophils’ average CD64 expression was significantly increased by ~3 times, whereas vehicle control injection did not induce changes in neutrophil CD64 expression (Figure 3e). CD64+ neutrophils increased from ~20.8% (Figure 3f) to ~47.5% (Figure 3g) in 10 min after TNFα injection.

Conventional flow cytometry was also used to assess the CD64 expression on blood neutrophils in TNFα-induced peritonitis (Figure 3h–j). We found that the CD64 expression on Ly6G+ neutrophils was significantly increased 10 min after TNFα but not after vehicle control injection (Figure 3h). Interestingly, the signal-to-noise ratio of TNFα-induced CD64 upregulation in the conventional flow cytometry assessment was less than the IVFC measurement (Figure 3e), possibly due to neutrophil activation induced by the invasive blood collection. Representative dot plots showed that CD64+ neutrophils increased from ~20.0% (Figure 3i) to ~46.1% (Figure 3j) in 10 min after TNFα injection, consistent with our IVFC assessment (Figure 3f-g).

### 3.4. More Neutrophils in the Circulation after CD18 Blockade in TNFα-Induced Peritonitis

**Figure 4:**
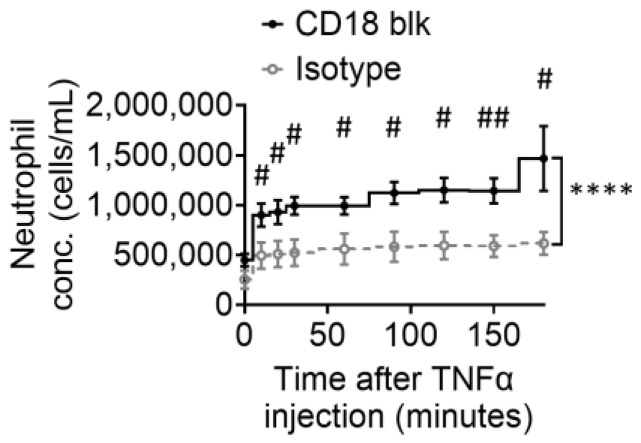
Neutrophil dynamics after CD18 blockade in TNFα-induced peritonitis. Dynamic circulating concentrations of LysM-EGFP+ leukocytes (mostly neutrophils) within 3 h after the i.v. injection of CD18 blockade antibody or isotype control and the *i.p.* injection of TNFα or vehicle monitored by IVFC. IVFC measurements were performed at minutes 0, 10, 20, 30, 60, 90, 120, 150, and 180. Mean ± SEM. n = 6 mice. **** *p* < 0.0001 according to the two-way ANOVA test. ^#^ *p* < 0.05, ^##^ *p* < 0.01 according to multiple unpaired *t*-tests.

CD18, or β2 integrin, is critical for neutrophil recruitment during inflammation [40,41,42], including peritonitis [20]. Here, we blocked CD18 using antibodies and used IVFC to monitor the dynamic changes in circulating neutrophils in TNFα-induced peritonitis (Figure 4). As expected, we found that, after CD18 blockade, the neutrophil concentration in circulation increased twice compared to that in isotype control, implying that more neutrophils are retained in the circulation after CD18 blockade, and that CD18 is required for neutrophil peritoneal recruitment.

## 4. Discussion

In this report, we demonstrated peritonitis-induced neutrophil recruitment on a real-time basis through IVFC. This technique enables the monitoring of the same population of cells in their native environment continuously and for a long period. Moreover, IVFC allows multiple measurements on a single animal over time, thereby minimizing the variation among individuals and the number of animals used. We used LysM-EGFP mice, in which most of the EGFP+ blood cells are neutrophils (Figure 2b). Alternative mouse strains to monitor neutrophils may include MRP8-cre-EGFP [43] and Ly6G-cre-tdTomato [44] mice. In MRP8-cre-EGFP mice, EGFP expression is specific in neutrophils but is too low to detect in the IVFC based on our experience [39]. Ly6G-cre-tdTomato mice have tdTomato specifically expressed in neutrophils. The in vivo imaging of tdTomato+ neutrophils was carried out [45]. Thus, this mouse strain will be a great alternative to monitor neutrophil dynamics in mice. IVFC can also be used to monitor other leukocytes. Previous studies monitored patrolling monocytes using CX3CR1-EGFP mice [30], neutrophils using LysM-GFP mice [30], and regulatory T cells using Foxp3-EGFP mice [27]. Thus, reporter mice are critical for IVFC studies. Besides reporter mice, antibody-based or ligand-based labeling might also work in IVFC. A previous study used folate-FITC and folate receptor antibodies to label folate-receptor-positive lymphocytic leukemia cells and lung carcinoma circulating tumor cells [26]. Previous studies showed that the Ly6G antibody could be used to label neutrophils in intravital imaging [33,46]. We used the PE-labeled CD64 antibody to monitor CD64 expression changes on neutrophils upon TNFα induced peritonitis (Figure 3), demonstrating the feasibility of labeling immune cells using antibodies and monitoring them using IVFC.

Compared to the previous study, monitoring neutrophils using LysM-GFP mice [30], our study has strengths and weaknesses. The previous study used a custom-build imaging system integrated with an optical phase-locked ultrasound lens (450 kHz resonance) and a galvo scanner to achieve microsecond-scale axial scanning, which can capture 3D images of neutrophils traversing through blood vessels at ~30 Hz and cross-sections of neutrophil images at ~2 kHz, allowing quantifications of cell deformability and cell–vessel wall interactions. However, the high cost of an optical phase-locked ultrasound lens and a galvo scanner limits its use. Other IVFC studies have a similar issue because most IVFC platforms are custom-built instruments, limiting access to IVFC in most biomedical studies. In contrast, our method can be used in any commercially available scanning microscope, including confocal or multi-photon microscopes, whether upright or inverted. Meanwhile, the raw data of our method are line-scanning 2-dimensional (x and t) images, which are much smaller than the 3-dimensional (x, y, t) or 4-dimensional (x, y, z, t) images and allow the recording of multiple mice for a longer period to obtain statistics required for disease study. Due to the importance of monitoring immune cell dynamics, IVFC is a useful method for understanding disease progression and the effect of any therapies. Our method greatly expands the use of IVFC in immunological research.

In our study, we used the vessels in the ear skin to noninvasively detect circulating cells, the same as in most previous IVFC studies [21,22,23,24,25,26,27,28,29,30] and studies monitoring neutrophil recruitment/swarming behaviors [47,48]. However, other skin vessels, such as the footpad [49,50,51], can also be used for intravital imaging and IVFC. Since our technique is based on scanning microscopy, the penetration depth (up to 200 μm for multi-photon microscopy and up to 100 μm for confocal microscopy) and depth of field (thickness of optical sectioning, <50 μm) limited the depth and diameter of vessels we can use for accurate measurements. For example, the tail vein would be too deep (~300 μm), and the diameter (~300 μm) would be too big for our assays.

## 5. Conclusions

In conclusion, we described a method adapting an intravital microscope for IVFC and developed an algorithm to process the data automatically. Using this IVFC method, we quantified the blood flow velocity and cell concentration in circulation. To the best of our knowledge, we are the first to report the noninvasive monitoring of circulating neutrophils and to show neutrophil dynamic changes in the TNFα-induced peritonitis mouse model. We showed an improvement in detection sensitivity compared to conventional flow cytometry. Thus, we provide a method to monitor immune cell dynamics during inflammatory diseases. Compared to conventional custom-built IVFC, our method has a wider range of adaptability, allowing more investigators to use it in their studies. The dynamics of immune cells will provide time resolution to better understand dynamic immune responses during diseases. Our method can be extended to monitor other immune cells, such as different T cell subtypes, B cells, natural killer cells, and other rare immune cells.

## Data Availability

All data are available at Harvard Dataverse https://doi.org/10.7910/DVN/31IDF4.
